# Unsuccessful Treatment of Aæsthesia by Cold

**Published:** 1855-01

**Authors:** 


### UNSUCCESSFUL EMPLOYMENT OF ANÆSTHESIA BY COLD.

(Cases under the care of Mr. Critchett and Mr. Walton.)—The employment of cold as a means of preventing the pain of operations has been repeatedly advocated in our columns, and we, therefore, feel called upon to report prominently instances of its failure. Two such have occurred during the past week. In the first, the patient was a woman, under the care of Mr. Walton, in St. Mary's Hospital, from whom it was wished to remove a fatty tumor on the abdominal wall. The tumor was subcutaneous, and felt quite as loose as such tumors generally are ; it had a size of about an adult fist, somewhat flattened. Nearly an hour was wasted in unsuccessful attempts to freeze the skin, but as this was due, of course, to mistakes in manipulation, it should not be charged against the process. At length, a mixture, properly made, was applied, and in about four minutes the requisite area of skin was frozen, as white and hard as could be wished. Without the loss of a moment's time, Mr. Walton made a deep incision through the whole required extent of skin into the tumor. This gave no pain. The tumor was seized at once, and forcible enucleation attempted. It could not, however, be extracted so easily as had been expected, and adhesion, both to the skin and the deeper parts, required to be divided by the knife. At one part, where it appeared to have been pressed upon by the edge of the woman's stays, the adhesions between the tumor and the skin were very close, and a careful division was needed. The operation lasted perhaps altogether about four minutes, and during the whole of that time, except the first cut in the skin, the patient was making loud cries and protestations of pain. It should be stated, that she was a remarkably quiet person, and one who did not complain for little.

The above operation took place on Wednesday ; and on the Friday following we witnessed an almost similar one in the theatre of the London Hospital. Mr. Crichett's patient was a man of middle age, and the tumor was a fatty one, about the size of a large fist, and situated beneath the skin in the upper part of the front of the thigh. The freezing of the skin was very complete, nearly five minutes had been occupied in the process, and the incision into it appeared to be quite painless. The tumor had, however, rather intimate adhesions, more especially to the integuments ; and the man complained much of almost every touch of the knife excepting the first.

We had witnessed before the above several cases of partial failure in the case of cold, but were inclined to attribute them somewhat to timorousness in its use ; in these, however, it was fairly and sufficiently used. Their evidence seems clear to the effect, that, unless the tumor be so loose, that almost instantaneous enucleation can be performed, a painless operation must not be expected. The anæsthesia does not extend at all deeper than the skin ; and even in it recovery of sensibility is so rapid during the manipulations, that the division of adhesions to its under surface will not be painless unless made without a minute's delay. There are, doubtless, a large number of cases in which, despite of these drawbacks, anæsthesia by cold may be made very useful ; but the Surgeon must always be careful not to promise to his patient a painless operation. As it regards the excision of tumors, it will probably, in a few instances, be completely successful, and in many others sufficiently so to afford a good pretext for avoiding the use of chloroform. It is, perhaps, adapted best of all for use in the very painful operations which it is so frequently necessary to perform on the fingers and toes. Here it can be applied from several sides at once, and a more complete and a less transitory degree of anæsthesia produced.—*Medical Times & Gazette.*

